# Perceptions of the Agency and Responsibility of the NHS COVID-19 App on Twitter: Critical Discourse Analysis

**DOI:** 10.2196/50388

**Published:** 2024-02-01

**Authors:** Dan Heaton, Elena Nichele, Jérémie Clos, Joel E Fischer

**Affiliations:** 1 School of Computer Science University of Nottingham Nottingham United Kingdom; 2 Lincoln International Business School University of Lincoln United Kingdom

**Keywords:** COVID-19, information system, automated decisions, agency metaphor, corpus linguistics, decision-making algorithm, transitivity

## Abstract

**Background:**

Since September 2020, the National Health Service (NHS) COVID-19 contact-tracing app has been used to mitigate the spread of COVID-19 in the United Kingdom. Since its launch, this app has been a part of the discussion regarding the perceived social agency of decision-making algorithms. On the social media website Twitter, a plethora of views about the app have been found but only analyzed for sentiment and topic trajectories thus far, leaving the perceived social agency of the app underexplored.

**Objective:**

We aimed to examine the discussion of social agency in social media public discourse regarding algorithm-operated decisions, particularly when the artificial intelligence agency responsible for specific information systems is not openly disclosed in an example such as the COVID-19 contact-tracing app. To do this, we analyzed the presentation of the NHS COVID-19 App on Twitter, focusing on the portrayal of social agency and the impact of its deployment on society. We also aimed to discover what the presentation of social agents communicates about the perceived responsibility of the app.

**Methods:**

Using corpus linguistics and critical discourse analysis, underpinned by social actor representation, we used the link between grammatical and social agency and analyzed a corpus of 118,316 tweets from September 2020 to July 2021 to see whether the app was portrayed as a social actor.

**Results:**

We found that active presentations of the app—seen mainly through personalization and agency metaphor—dominated the discourse. The app was presented as a social actor in 96% of the cases considered and grew in proportion to passive presentations over time. These active presentations showed the app to be a social actor in 5 main ways: informing, instructing, providing permission, disrupting, and functioning. We found a small number of occasions on which the app was presented passively through backgrounding and exclusion.

**Conclusions:**

Twitter users presented the NHS COVID-19 App as an active social actor with a clear sense of social agency. The study also revealed that Twitter users perceived the app as responsible for their welfare, particularly when it provided instructions or permission, and this perception remained consistent throughout the discourse, particularly during significant events. Overall, this study contributes to understanding how social agency is discussed in social media discourse related to algorithmic-operated decisions This research offers valuable insights into public perceptions of decision-making digital contact-tracing health care technologies and their perceptions on the web, which, even in a postpandemic world, may shed light on how the public might respond to forthcoming interventions.

## Introduction

### Background

The agency of automated decision-making algorithms is a long-standing debate in academic research [1-3]. Decision-making algorithms can mitigate human errors or inaccuracies [4-7]. However, when they operate decisions in lieu of individuals, the algorithms can be seen to develop a social agency and be perceived as having humanlike characteristics [8,9]. When decision-making algorithms do not perform their assigned tasks as expected, investigating algorithmic agency can avoid additional problems, such as reinforcing bias or outcomes and undermining trust in automated decision-making systems [10-13].

An exemplary decision-making algorithm that has had significant societal impact is the National Health Service (NHS) COVID-19 app [14,15], which was used to mitigate the spread of COVID-19 in the United Kingdom by tracing contact with infected individuals and notifying people to self-isolate. Launched in September 2020, that is, 6 months after COVID-19 began to spread in the United Kingdom, this app gained public attention due to an array of issues and concerns [16-18], with social media websites such as Twitter voicing the views of many users. Several studies have investigated the sociological and epidemiological impact of the app [15,19-21], yet a gap persists with regard to how the app has been discussed on Twitter, specifically.

Agency expressed on social media can be investigated in multiple ways, such as through interview or observation [22,23]. We purposefully used corpus linguistics (CL)–informed and critical discourse analysis (CDA)–informed approaches to examine the relationship between grammatical agency and social agency [24]. Grammatical agency, or transitivity, can show whether an entity is presented actively performing an action or passively having an action performed onto them [25]. Deconstructing the agency of decision-making algorithms in the discourse can shed light on the perceived power relations between entities [26] and how these can ultimately indicate social actors in discourses [27].

To address the lack of examination of grammatical agency and transitivity in social media discourse, we uncovered the social agency of decision-making algorithms by examining tweets mentioning the NHS COVID-19 app. Specifically, we contributed to answering this overall question: How is social agency discussed in social media public discourse dealing with algorithmic-operated decisions when the artificial intelligence (AI) agency behind specific information systems is not openly disclosed? We added to the discussion around this answer by focusing on the following two research subquestions:

1. How is the NHS COVID-19 app presented on Twitter, especially with regard to social agency, and how society has been impacted by the deployment of this system?
2. What do these presentations of social agents communicate about the NHS COVID-19 app’s responsibility to process information as perceived by the authors of the social media public discourse (ie, Twitter data) analyzed in this study?

By applying 2 methodological approaches, CL and CDA, underpinned by social actor representation (SAR) [27], we examined the use of the word “app” in context, which can be understood, as we will show, as a common grammatical subject of perceived agency. Thus, our work shows how the social agency of the app is implied or established through how users present it via grammatical constructions. Therefore, this study aimed to investigate the agency of the NHS COVID-19 app and how it was perceived by the public via its presentation on Twitter. Focusing on the relationship between grammatical and social agency and its impact on society, this study sheds light on the responsibility and blame attributed to the app in processing health care–related information.

### Prior Work

To see whether Twitter users perceive the app to be a social actor, in its own right, or whether it is passively controlled, by the algorithmic-based AI system on which it operates, this section reviews the existing literature on the relationship between grammatical and social agency and agency and decision-making algorithms and gives an overview of the NHS COVID-19 app.

#### Grammatical and Social Agency

When defining agency, Gallagher [28] stated that it is a clear feeling of control and suggested that it impacts human consciousness. Silver et al [29] stated that a sense of agency also encompassed the responsibility felt due to actions undertaken and the effects they have. Therefore, social agency could be uncovered by examining grammatical agency [24-26].

Grammatically, Leslie [25] defined an agent as an entity with an internal source of energy through which it exerted force to carry out the activities referred to in the text. Expanding on this, Richardson et al [24] stated that agency in linguistics is often explored by examining how it is emphasized, manipulated, or concealed. As such, transitivity analysis—the examination of agency in the text—examined the use of active and passive voice or nominalization, where verbs were the word class converted to nouns. Accordingly, choices revealed the attitude and ideology of the language user or perceived agent [30]. In addition, research has shown that passive constructions tend to remove agency from the subject or dilute its impact [31]. Especially, when the subject was absent from the clause, the implied responsibility shifted [26]. Arguably, this refers to decision-making power, which was investigated in this study.

Alternatively, the agency can be conveyed through lexical choices. For instance, Morris et al [32] suggest that “people believed acceding trajectory evokes the impression of high animacy, which would be caused by enduring internal property, i.e. the volitional action” (eg, “the NASDAQ fought its way upward”). In contrast, “the descending trajectory suggests inanimacy, as a result of lack of external forces” (eg, “stocks drifted higher”). This phenomenon, called the agency metaphor, constituted the focus of this analysis, as well as transitivity, as they both communicate the capacity or potential to finalize decisions.

#### Agency and Decision-Making Algorithms

The last decade has witnessed an increased focus on the perceived social agency that decision-making algorithms have [1]. During this period, decision-making algorithms were deployed to solve societal problems, such as corruption, unethical practices, different interpretations, inaccuracies in assessments, and, perhaps most notably, inefficient decision-making [4]. Although the legal treatment of decision-making algorithms received significant attention in the literature [33-35], the social agency of these algorithms still seems underexplored. Nevertheless, given the increasingly crucial role that algorithms play in public life, it is important to unveil the relationship tying algorithms, agency, and autonomy together [36].

Among researchers who looked into the specifics of social agency and whether decision-making algorithms are perceived to have it, Rubel et al [36] defined several key ways in which issues of agency, autonomy, and respect for humans may be at odds with algorithmic decision-making. More specifically, algorithms may create rules that are impossible to follow and may not provide a rationale for decisions. Moreover, they may fail to provide room to appeal to outcomes, may not respect interpersonal boundaries, and may allow those deploying them to avoid repercussions in terms of finalizing decisions.

Other studies specifically examined the impact of decision-making algorithms and their perceived autonomy. For example, Lamanna and Byrne [37] focused on health care–related applications for decision-making algorithms and found that automating decision-making processes would be dehumanizing but argued that including algorithms would aid in stressful decision-making for medical professionals. However, this idea is not only applicable to medical studies. For instance, Riegler [38] investigated the impact of using decision-making algorithms to aid in stressful situations, but within the context of autonomous vehicles.

The social power that algorithms hold was investigated by Beer [11], who argued that although concerns regarding agency can be somewhat complex, algorithmic power is generally assumed to imply some sort of agency. Studies looking specifically at concerns surrounding the merging of human and algorithmic agencies [8,9] have questioned whether to make algorithms more humanlike or humans who are similar to algorithms [39].

Interestingly, humans may oppose the decision-making algorithms. According to Mahmud et al [40], the reasons behind algorithm aversion can be extracted from the existing literature and categorized as high level (societal), algorithm related, and factors concerning an individual. Within the algorithm factors category, one was the anthropomorphic presentation of an algorithm, along with its complexity, understandability, accuracy, and ability to learn. Similarly, Grange [41] indicated the use of machine learning or “black-box” techniques as a tool to automate decisions. Because humans may be unaware of how algorithms are designed to operate, it is critical that the perceptions of those impacted by algorithmic-based decisions are taken on board throughout the design, implementation, and review processes.

Despite the explanations provided, an agent may be systematically judged differently when they are perceived as artificial rather than human. For example, Feier et al [11] found that decision makers can rid themselves of guilt more easily by delegating to machines than to other people, thus showing that the availability of artificial agents could provide stronger incentives for human decision makers to delegate morally sensitive decisions. Therefore, decision-making algorithms can deflect responsibility, and thus blame, from human decision makers to artificial intelligence–operated systems.

Considering this, it may be worth questioning whether an algorithm can be presented as having the same agency as humans. In addition, the impact that this sort of presentation might have can provide complementary insights into this topic.

#### The NHS COVID-19 App

The NHS COVID-19 app, the contact-tracing algorithmic-based system created by Serco on behalf of the UK government to track active cases of COVID-19, has impacted the United Kingdom on multiple levels since its launch [14]. The app is available on mobile phones and uses exposure logging, as developed by Apple and Google [42]. This technology allows the app to send alerts using a randomly generated ID number when the user is close to another app user who has logged a positive COVID-19 test. Despite its scientific-based intended functionality, its users reported issues regarding backward incompatibility, incorrect alerts, and false-positive tests. Such unexpected technical problems meant that users had to self-isolate for 10 days even when the result was incorrect, with inadvertent consequences on their income and well-being [15].

Despite the UK government encouraging its adoption, the uptake of the app was less than expected at 20.9 million downloads between September and December 2021, with 1.7 million notifications being sent out in England and Wales [43]. According to Wymant et al [19], every 1% increase in the number of app downloads leads to a 0.8% to 2.3% reduction in the number of COVID-19 infections, with their findings suggesting that anywhere between 100,000 and 900,000 cases were averted because of the information inputted by users into the system. However, Mbwogge [17] claimed that a symptom-based contact-tracing system failed to meet the testing and tracing needs in the United Kingdom, which is further evidenced by the fact that cases and deaths relating to COVID-19 increased to be the highest in Europe.

Perhaps because of its technical challenges, a growing number of research projects have investigated the public attitudes toward digital contact tracing in the United Kingdom. Williams et al [44] interviewed 27 participants using web-based videoconferencing before the release of the COVID-19 app in the United Kingdom and found the response to be mixed and heavily influenced by moral reasoning. The analysis revealed 5 themes: lack of information and misconceptions surrounding COVID-19 contact-tracing apps; concerns over privacy; concerns over stigma; concerns over uptake; and contact tracing as the “greater good.” Samuel et al [45] conducted 35 semistructured qualitative interviews in April 2020, showing interviewees’ views on the potential of the app for contact tracing. The participants showcased a range of misconceptions and worries. However, as there was no follow-up to this study, it was impossible to discover which participants would then choose to download the app once it was launched in September 2020. These insights shall inform our investigation of the impact of the NHS COVID-19 App in British society throughout the pandemic and the perceptions of this system by its (intended, actual, or former) users.

This possible evolution of attitudes toward the app was instead monitored by Dowthwaite et al [16], who surveyed 1001 UK adults and found that half of the participants had installed the app, with 60% of them claiming to comply with it on a regular basis. They also found that there were issues surrounding trust and understanding that hindered the effective adoption of the app. A follow-up analysis showed that there were statistically significant correlations between lower trust among nonusers, many aspects of the app, and the wider social and societal context [46]. A year after the app was launched, Pepper et al [2] identified 5 main themes during follow-up interview discussions: flaws in the app, usefulness and functionality affecting trust in the app, low trust in the UK government, varying degrees of trust in other stakeholders, and public disinterest. According to the study results, these factors contributed to a drop in compliance over time. Similar findings were proposed by Paucar et al [18], who stated that responsibility and trust made the app better accepted by the public. Even though these were always relevant, other factors, such as fear of infection, were contextual and time dependent. Arguably, this will be relevant when examining the presentations of social agents who tweeted about such an app system and its functionality as perceived or evaluated by its self-proclaimed users or experts.

For instance, in July 2021, when the relaxation of government restrictions led to an increased number of positive COVID-19 cases in the United Kingdom, media scrutiny of the app intensified because of the numerous notifications sent through the app [47]. As a result, this impacted the public’s perception of the app and the pejorative blend “pingdemic” was coined [48]. This exemplifies the considerable impact that the deployment of the NHS COVID-19 App has had on British society and how this was reflected by the media and social media and the terminology they used.

To date, one study has examined tweets relating specifically to the app in the United Kingdom. Heaton et al [49] found, using a mix of computational linguistic tools, a general sentiment trend saw positivity detected within tweets from September to November 2020 only for tweets categorized as more negative in December 2020 and January 2021. Positive sentiment rose again in February and March 2020, dipping slightly in April, but rising again in May and June. Tweets were deemed less positive in July. Prominent topics included how the app works, obtaining the app, and the development of the app. Trust and fear were the most frequently detected emotions. However, what could not be ascertained through this research was how Twitter users specifically presented the app in terms of its agency or impact. Although it has been established that there is research interest in digital contact tracing from a sociological and epidemiological standpoint [2,16,18,20,21], a gap in the presentation of the app itself was detected, which our contribution aims to address.

## Methods

### Data

Data were collected with the aid of Twitter for Academic Purposes Application Programming Interface. Twitter was chosen as a data source because of the large amount of real-time data available [50]. In addition, Twitter data could be preprocessed before analysis [51], lending itself well to exploratory analysis principles [52].

Following the best practices recommended in social media research literature, we did not include any screenshots of tweets that may later identify their author, without obtaining consent. Instead, as part of the data-cleaning process, tweets were anonymized and only short extracts from tweets were reported verbatim (therefore, including typographical or grammatical inaccuracies). The project design was approved by the ethics committee of the university department. The data were pseudonymized during extraction, with a unique number generated to refer to each tweet. Stopwords were removed from the data set using Gensim, along with the removal of all long and short URLs and the indication “RT” (retweet) at the beginning of any tweet. Twitter handles that appeared within the tweets were also redacted using Gensim, for anonymity.

With permission, this study used the same data set as Heaton et al [49], as data had already been collected relating to this topic. Data extraction was performed using the Tweepy module in the Python programing language [53]. The key search criteria for this were tweets containing “@NHSCovid19App,” which is the official Twitter handle for the United Kingdom’s contact-tracing app and the related hashtag “#NHSCovid19App.” The reason for this choice was to ensure that tweets were directly related to the experience of the contact-tracing app itself, rather than to the wider NHS Test and Trace system or the COVID-19 pandemic generally. Although key parts of the discourse may not be revealed through this search term alone, it provides a starting point for investigating the views expressed regarding the app.

In total, 180,281 tweets (1,797,052 words) were collected from September 23, 2020, the day before the app was launched in the United Kingdom, to July 31, 2021. Furthermore, a second data set was collected using the search term “pingdemic” to capture relevant tweets relating to the surge in self-isolation notifications in July 2021. This data set contained 36,022 tweets (831,579 words). Subsequently, tweets were condensed to remove advertisements from the data set, resulting in a final corpus of 118,316 tweets over an 11-month period. The data were sourced from the United Kingdom, and only tweets in English were selected. Therefore, the analysis investigated views expressed only in English.

### Ethical Considerations

Despite these advantages, complex ethical considerations are imperative when scraping Twitter data for analysis. Although tweets are public (by default), Twitter “data” is not intentionally provided by users for the purposes of research, yet gaining explicit informed consent to use their tweets for research is practically infeasible [54]. To align with the best practices in social media research, we anonymized tweets during data cleaning and shared only concise verbatim excerpts. The project design was approved by the ethics committee of School of Computer Science at the University of Nottingham (approval number CS-2020-R33).

### Corpus Linguistics

CL was deemed as a suitable approach to answer our research question because of its ability to analyze large data sets, using specialized pieces of software to uncover patterns’ relative efficiency [55]. CL takes the idea of further investigating the corpus (ie, the body of data collected to analyze) through a multitude of different language-focused perspectives. For example, CL can facilitate diachronic comparisons across corpora by focusing on lexical use [56]. Because of its capacity to identify language patterns in data sets containing hundreds of words [57-59], CL has been frequently deployed to analyze social media [60-62]. We also chose CL as it allows for the comparison of multiple corpora, identifying trends and patterns that distinguish multiple data sets. Thus, CL is particularly helpful when comparing data from different periods, as in this study.

With the aid of CL-computerized tools, we focused on collocation, that is, the co-occurrence of 2 or more words within a predefined word span [55]. When using frequency as the sole measure, Baker [63] stated that it might not be possible to verify whether a co-occurrence is a true reflection of a semantic relationship, that is, a connection based on word meaning, or whether chance played a part. Instead, statistical significance measures, such as LogDice or Log Likelihood, are useful indicators of lexical and grammatical associations between textual elements as well as themes [64]. In this sense, concordances help identify collocations, as they can show how adjacent or in close vicinity the related words are together [65].

The CL software used to undertake this analysis was The Sketch Engine [66], which was chosen for practical and analytic reasons. Indeed, it is freely available to many academics, it allows the upload of ad hoc corpora, and it provides The Sketch Engine a series of reference corpora that can be used for comparisons.

The analyses performed in this study were conducted in different stages. First, keyword analysis was used to distinguish keywords in the discourse using the embedded English Web 2020 (enTenTen20) [67] as the term for comparison. EnTenTen20 has over 36 billion words of specifically internet texts, including social media, and so acts as a suitable reference corpus. In addition, keyness scores were generated by comparing the frequency of words in the target corpus to the frequency of words in the reference corpus. This allowed us to examine the key characteristics of the corpus compiled, proving an overview of the tweets collected for the analysis.

Second, concordance lines featuring “app,” as a potential social actor, were examined to prompt the collocation analysis, which was accomplished following different directions. To ascertain active constructions, our collocation criteria were “app” and one verb to the right (R1). To ascertain passive constructions, our search criteria was “by the app” and one verb to the left (L1). Passive constructions, such as “the app was used by many” and “the app has not been created” were included in the examination of verbs to the right (R1). Specifically, as they are passive constructions where the app is the object, they were removed from the active constructions and added to the passive construction category manually.

LogDice was considered as the statistical measure of collocational strength. LogDice compares the observed co-occurrence of words with their expected co-occurrence based on their individual frequencies. A high LogDice score indicates a strong association between 2 words, suggesting that they often appear together, whereas a low score implies a weaker association. LogDice was included as it not only measures the statistical significance of a collocation but also factors in the size of the subcorpus, making comparisons between subcorpora of different sizes easier.

To take advantage of this capacity, we split the corpus into 5 subcorpora that reflected the key moments in the evolution of the pandemic in the United Kingdom in chronological order:

- Period 1: app launch (September 2020)
- Period 2: early months (October-December 2020)
- Period 3: second national lockdown (January-February 2021)
- Period 4: later months (March-June 2021)
- Period 5: “Pingdemic” (July 2021)

We report the strongest collocates for each period, ranked by LogDice score.

We used a minimum threshold of 3 occurrences for the collocate to be significant enough to report; hence, the variation in collocates at each time.

### Critical Discourse Analysis

Finally, from this, CDA was applied to examine agency and social action as expressed in the (concordance) lines, where “app” appeared as a keyword in context. In this study, CDA was used complementarily to The Sketch Engine CL analysis tool [66] to pinpoint different perspectives and meaning shades, referring to the various subtle nuances or different shades of meaning that a word or phrase can convey [68]. Therefore, these approaches were deemed especially effective together, as accomplished in studies [69-71] with similar purposes to this analysis.

CDA is an interpretative qualitative approach to text analysis that draws on related theoretical frameworks [72-74]. Overall, it can be used as a tool to better understand meanings implied through textual context [75-77]. Several studies have demonstrated the benefits of using CDA on Twitter discourses [78-80]. This fits with our data-driven approach to analysis in an attempt to answer our research questions regarding the presentation of the app and its impact on British society.

To successfully analyze the relationship between grammatical and social agency, we underpin our use of CDA within the theoretical framework of SAR, which is drawn from Social Action Theory. According to Social Action Theory, people create society, institutions, and structures [81]; hence, examining social actions can provide an explanation for human behavior and societal change [82], including the app users’ perception, which we intend to focus on in this study.

More specifically, SAR examines how grammatical structures convey social agency; for example, active or passive constructions and transitivity structures can be used to communicate who social actors are in a discourse of interest [27]. Similarly, syntactical features, sentence structures, and verbs within tweets give us an indication of how users perceive the app to be responsible for processing their information.

Multiple concepts can be considered as key when it comes to sociosemantic categories for analyzing social actors [27]. Among them, the removal of grammatical agents is called excluding. Conversely, when clues are left as to who the agent is, this is called backgrounding. In addition, actors can be personalized through word choices pertaining to the semantic nature of being “human” or impersonalized. Moreover, examining agency metaphors, previously outlined in this paper [32], can signal further personification of nonhuman entities. All of these aspects are especially relevant to this study because they imply humanlike perception, possibly indicating whether responsibility for consequences is attributed.

At the same time, social actors could be a group of people (genericized) or represented as single individuals (specified). In this sense, indetermination occurs when social actors are not specified (such as “someone”), whereas determination occurs when their identity is made known. All of these representation structures play a role in indicating the social and power dynamics within discourse, as shown in other Twitter case studies that used CL, CDA, and SAR [83-85]. In this analysis, tweets were examined in terms of the determination and indetermination they carry, as to how they deflect or attribute responsibility.

By analyzing all these characteristics in this Twitter discourse, we intended to identify common presentations of the NHS COVID-19 App, ultimately displaying how power relations are communicated in real-life data dealing with algorithmic-operated decisions, even when the mechanisms are not fully clear. After establishing these, we identified similar semantically related thematic groups (as seen previously in Razis et al [86] and Kitishat et al [87]) that could aid in the analysis of the presentation and perceptions of the app over time.

## Results

### Keyword Analysis

Table 1 shows the top 10 words with the highest keyness score when compared with EnTenTen, 2020 (all scores to 2 decimal places). The word with the highest keyness score was app, which supported our initial thinking that this would play a dominant role in the discourse because all tweets collected for this study included the expression “NHSCovid9App” to intentionally focus on discussions revolving around this system. With this in mind, we proceeded with the analysis as planned.

**Table 1:** The top 10 words with the highest keyness score.

Item	Relative frequency (per million)			Score
	Focus corpus	Reference corpus		
app	13,387.27	63.94	82.27	
nhs	5427.78	15.62	47.81	
download	3597.18	61.05	22.96	
covid	1846.74	4.39	18.65	
serco	1556.30	0.26	16.52	
trace	1613.03	94.14	14.84	
isolate	1355.90	4.08	13.99	
test	3302.05	159.01	13.14	
qr	1236.71	2.22	13.08	
downloaded	1351.74	11.42	13.03	

### Presentation of the App: Timeline Overview

This section presents a timeline of the changes in the grammatical presentation of the app along with the potential social implications that this had. As part of these results, we found that “be” and “have” were frequently occurring collocates of the “app.” Upon manual inspection of the tweets containing them, most were found to be auxiliary verbs. Whenever this was the case, they were considered as multiword expressions and analyzed on the basis of their overall meaning because they conveyed links between agency and responsibility together. First, we looked at the frequency of active and passive verbal constructions including “app.” This overview is shown in Table 2, where the information system features actively in 97% of clauses. However, active and passive constructions alone do not necessarily provide a full account of how the app is presented in the discourse. For example, the app could be presented actively, yet it could carry limited social agency (eg, “you self isolate when the app pings you even though you don’t have to”). To avoid misinterpretation, we combined CL and CDA.

**Table 2:** Frequency of active and passive presentation of app.

Period	Active	Passive	Total
September 2020	3700	138	3838
October-November 2020	1935	65	2000
January-February 2021	1396	36	1432
March-June 2021	740	5	745
July 2021	6108	122	6230

### Period 1: App Launch (September 2020)

The launch month of the app saw 17,759 instances of the word “app,” which was the highest engagement recorded across any month included in the corpus.

#### Active Presentations in September 2020

In September 2020, there were 3700 instances of active app presentations. The strongest 20 collocates are presented in Table 3. Many of these active presentations evaluate the app as underperforming, especially constructions containing “do” (LogDice: 9.42). Tweets stated that the app “doesn’t do its job” or “doesn’t work.” In this sense, such instances reflected public perceptions of the app, which were frequently deemed dysfunctional.

In September itself, “app” and “say” (LogDice: 8.07) frequently co-occur in tweets which discuss the app presenting information that users struggle to understand. For example, one tweet questioned why the app “says” they “are in a medium risk area.” Another tweet stated that, despite going elsewhere, the app “said” they were “still at home.” Occasionally, users complained that it “says nothing.” Tweet authors’ use of the verb “say” suggests that the app behaved (or was perceived as behaving) like a human; hence, it is an illustration of personalization.

Another strong collocate of “app” was “tell”(LogDice: 7.90). This had semantics similar to “say.” However, “tell” was mainly used to express that the app was instructing a user to self-isolate, in both actual scenarios (ie, upon entering test results into the app, the app “tells” them “to isolate and get a test”) and hypothetical scenarios (eg, the app “told” them they “had to isolate even though their boss would not allow them to without symptoms”). In other instances containing “tell,” users questioned the reliability of the app, for instance asking whether anyone believed “a word this app tells u?” Similarly, another user was confused about “what this app is telling [them]??” Although comparable with “say” semantically, it could be argued that the pragmatics of “tell” were different. For instance, “tell” acted imperatively when “telling” users “to stay at home.” This constituted the personalization of the app. These examples could also be considered agency metaphors, as “tell” implied more volitional action as an imperative compared with “say.”

Another way in which the app was presented actively was when users wrote that it “needs” something (LogDice: 7.50). In this month, users frequently tweeted about the operating system requirements for the app to function on mobile devices, whereby the app “needs ios 13.5” and “needing current ios updates or [it] won’t work.” The “needs” of the app not only presents it actively but also gives it humanlike characteristics, providing other examples of personalization, and hence a fuller account of the app’s public image.

“Allow” also strongly collocates with “app” (LogDice: 7.35). In these instances, users discussed the function of the app and the permissions that the app granted. For example, “the app allows [them] to enter one postcode only,” causing issues to people living and working in different areas. Interestingly, the user directed this grievance to the app itself, giving the impression that the app had social agency. Other occurrences of “allow” involved questions, for instance, asking whether the app will “allow” users to report themselves as testing positive, even when they are not. Another questioned if the app “allow[ed] for manual check in.” These questions from Twitter users reiterated concern for the app’s implied agency and could potentially be seen as additional examples of personalization, expressing public attitudes of uncertainty and worry about not being able to use the app.

“Think” is another strong collocate (LogDice: 7.24). Occurrences of this active presentation complained about the app’s performance and accuracy. For example, when visiting different places, the app could “think” that users were “still at home.” Another Twitter user reported deleting the app after getting a negative test back as there was no code to input the test and the app “thought” they “still had to isolate.” One question was whether the app “thought” they had been “at the old venue for all that time” when they checked into a new venue after several days. Despite these open queries and concerns, the users presented the app as being able to think and act for themselves. In this case, personalization conveyed agency.

**Table 3:** Top 20 words ranked by collocational strength of “app” + one verb to the right (R1) in September 2020.

Rank	Collocate	Frequency	Collocate frequency	LogDice
1	do	567	23,295	9.42
2	be	1565	80,974	9.24
3	use	111	6766	8.43
4	work	108	7093	8.34
5	require	39	655	8.17
6	have	297	30,674	8.14
7	say	64	4011	8.07
8	tell	52	3357	7.9
9	fix	30	663	7.79
10	launch	25	724	7.5
11	need	47	4738	7.5
12	allow	23	815	7.35
13	know	44	5289	7.31
14	think	32	3154	7.24
15	install	21	871	7.2
16	seem	20	1221	7.03
17	develop	15	412	6.87
18	go	30	4620	6.87
19	let	19	1563	6.86
20	delete	17	1018	6.86

#### Passive Presentations in September 2020

The app is also presented passively on 3.59% (138/3838) of occasions this month, with collocates as shown in Table 4. These included discussions about whoever created the app. “Be” + “develop” was a multiword collocate of “app” (LogDice: 10.00). Some constructions were questions such as whether the app has “been developed by the nhs?” or the “app was developed by serco and [...] not the nhs.” These tweets are examples of the backgrounding of the entities that (supposedly) created the app. Instead, this presents the app as passive, yet important in the construction, as, despite the lack of grammatical agency, the focus is still on the app. This is closely linked to “be” + “design” (LogDice: 9.4). This discussion around the app’s intended function was subverted in some tweets; for example, “this app is designed to control sheeple.” However, unlike the previously mentioned examples, these represented instances of exclusion and removed the agent from the construction. In these passive structures, Twitter users still discussed the app in a negative way, highlighting that the app’s functionality was deemed unsatisfactory by its (self-declared) users.

Similarly, the “app” collocated strongly with “be” + “run,” resulting in constructions containing the passivization of Serco. Examples included “but then the app is run by a private company” and “have heard this nhs app is run by serco?!” This again indicated that despite the passive presentation, users were still dissatisfied with the app.

Comparable instances portrayed the app passively, through a “has been” + verb construction (LogDice: 4.88) to state that the app “has been launched in england and wales after months of delay” or that “the government’s app has been designed by a dog.” Neither of these constructions indicated who was responsible for the launch or design of the app, thus exemplifying exclusion and using it to reiterate user dissatisfaction.

Other passive constructions, delivering a similar meaning, combined a verb and “by the app” and accounted for 44 of 138 occurrences in September 2020. “Recognize” (LogDice: 8.70) was mainly used regarding the inputted test results into the app. For instance, one user asked whether “only private tests will be recognised by the app” and another stated the simplicity of setting up code “recognised by the app.” In both short extracts, the app’s passive presentation removes agency and places it more with app developers. In terms of agency, “accept” (LogDice: 7.36) is similar to the data analyzed. For example, one user complained that the incorrectly formatted code was “not accepted by the app.” All of these instances reflected the app’s perceived lack of functionality, causing public criticism despite the passive presentation.

“Isolate” is another strong collocate of “by the app” (LogDice: 5.36). Tweet authors complained about being told to self-isolate using the app. One user, for instance, questioned liability if “notified of contact/need to isolate by the app.” Thus, the app appears to be less of a focus in the structure, and agency is removed through passivization and backgrounding. Therefore, the responsibility could possibly be transferred from the app to the user.

“Tell” (LogDice: 4.85), featured in complaints about people that they were being instructed by the app. An example was one user discussing a “person at my work” who had “just been told by the app to self-isolate and get a test.” This presented the app passively and paid limited attention to it, with the “person” being the central figure, although indetermined and genericized. As in the previous case, responsibility seemed to be deflected to the user “by” the app.

In summary, in September 2020, many tweets actively presented the app, especially when uncertain about how the app functioned or could assist its users. This was mainly accomplished through personalization, portraying the app as if it were human. In these cases, passive presentations prominently discussed the development of the app and attributed it to Serco, the NHS, or the UK government, deflecting the responsibility from the app to these organizations or app users.

**Table 4:** Top 6 words ranked by collocational strength of “by the app” + one verb to the left (L1) in September 2020.

Rank	Collocate	Frequency	Collocate frequency	logDice
1	recognise	5	350	8.7
2	accept	3	554	7.36
3	isolate	4	3137	5.36
4	track	3	2653	5.19
5	tell	3	3357	4.85
6	use	4	6766	4.27

### Period 2: Early Months (October-December 2020)

The first 3 full months after the app was launched saw 6237 tweets using the word “app.”

#### Active Presentations in October to December 2020

Active presentations of the app were seen 1935 of 2000 times, with the R1 collocates reported in Table 5.

“Use” (LogDice: 10.42) appeared most frequently in a duplicated tweet that had been sent from different regional NHS accounts. The text in question contained the structure “the app uses an algorithm to filter out false alarms.” Therefore, the NHS promoted the app as a positive social actor in contrast to the negative presentations put forward by several members of the public, as detailed earlier.

Similarly, many of the tweets using “say” (LogDice: 8.58), released over these 3 months (October-December 2020), were comparable to those published at the time of the app launch (September 2020). Among others, one user tweeted about discrepancies between the supposed ending to their self-isolation period, stating that their app “said that [their] self isolation will be ended on 25 dec 2020 at 23.59,” which was “different from what [they] have been told on text message and nhs website.” Here, the app was presented as actively informing the user, which constituted another example of personalization. Interestingly, the same user states that they have been “told on the text message and nhs website,” rather than be told by the message or by the website. This distinguished the app actively presented and other technological systems, appearing as vessels of information, rather than agents. These different presentations reaffirmed that the app was a social actor in this context.

“Tell” (LogDice: 8.55) was used in a similar way to “say,” similar to the tweets found in September 2020. An example of this included one user tweeting that their child had “received a notification on the track & trace app telling her to self-isolate” yet only for 2 days. Another user stated that the app “tells” them their “home is medium risk” despite living in a rural area with low COVID-19 infection rates. These examples indicated that the app was providing instructions and thus had a social agency.

Another strong collocate was “have”(LogDice: 7.02). Although used as an auxiliary verb in most constructions, there were occasions when it acted as the main verb to indicate possession (or a lack of possession). For example, one tweet discussed that their relative was recovering from cancer and expressed frustration that the app had not notified them even though they had been in contact with a positive case. Accordingly, the app “has one job” to keep their relative safe. This is a clear example of personalization due to the idea that the app is able to perform a job yet responsible for the safety and welfare of their relative. Similar active presentations featured “have” as an auxiliary verb, as when a user joked that the app “has decided to turn off contact tracing,” implying its autonomy and control.

When the app was presented as performing the opposite of its desired function, negation was used. A user complained that “the app has not alerted [them]” despite “living with someone who had tested positive” for the virus. Another user stated that their app “has not conducted exposure checks since 29 december.” Both examples placed the agency with the app, alluding that the app was responsible for its own shortcomings. With “someone,” this is an example of an indetermined construct, which further removed agency from the humans and placed it with the app.

On other occasions, where users wrote that the app “gives” them something (LogDice: 6.88), one complained that the app “gives [them] notification about people passing by [their] house,” while another joked that the app gave them “a 3 day stay at home order.” Another mused that the app was “giving the govt more control over our everyday lives.” In all of these occurrences, the app was presented actively through personalization, showcasing the perceived responsibility of the app for controlling users’ lives.

**Table 5:** Top 20 word ranked by collocational strength of “app” + one verb to the right (R1) in October, November and December 2020.

Rank	Collocate	Frequency	Collocate frequency	logDice
1	use	365	6766	10.42
2	say	70	4011	8.58
3	tell	61	3357	8.55
4	work	84	7093	8.25
5	be	703	80,974	8.12
6	do	154	23,295	7.64
7	have	129	30,674	7.02
8	show	12	1119	6.99
9	give	14	1920	6.88
10	allow	9	815	6.73
11	send	10	1134	6.72
12	install	9	871	6.7
13	seem	10	1221	6.68
14	store	15	2838	6.68
15	update	9	1443	6.43
16	develop	6	412	6.37
17	fail	6	446	6.35
18	keep	10	2083	6.34
19	crash	5	83	6.32
20	ask	8	1685	6.17

#### Passive Presentations in October to December 2020

When examining passive constructions, as shown in Table 6, passive presentations similar to the previous month can be seen. When focusing on “notify” (LogDice:7.87), tweets focused on hypothetical scenarios, with one tweet stating that they were not entitled to support should one be “notified by the app” as “they can’t identify you” and another that questioned the legal ramifications if one was “only notified by the app” and not test and trace as a whole. These examples discussed the legal and financial implications of the app directing someone to self-isolate. In both instances, the app was not a prominent part, hence, passivization, and the central focus was on the impact rather than the app.

In contrast, when “tell” was used in passive constructions (LogDice: 5.59), many of these accounts were direct first-person narratives by app users. For example, one “got told by the app [...] to isolate for 12 days.” Another explained they have “not been told by the app to isolate,” even after their family member tested positive. In these cases, the authors recounted that they were provided with a service by the app, backgrounding the importance of the system in the process. Instead, these accounts tended to focus on obtaining answers from humans that the app could not provide.

Some “have” constructions were passive too. For example, one user wrote that they were at risk as the app “has not been created to include old smartphones.” This passive construction implied that the app had been created by an unknown agent, thus exclusion. Although this passive construction removed some agency from the app, the fact that IT was mentioned explicitly in the tweet could still foreground the system as a social actor.

**Table 6:** Top 3 words ranked by collocational strength of “by the app” + one verb to the left (L1) in October, November, and December 2020.

Rank	Collocate	Frequency	Collocate frequency	logDice
1	notify	3	385	7.87
2	isolate	5	3137	5.69
3	tell	5	3357	5.59

### Period 3: Second National Lockdown (January-February 2021)

#### Active Presentations in January to February 2021

During this period, there were 1396 active presentations of the word “app,” as shown in Table 7. “Ping” (LogDice: 7.98) was used actively to mean notify, with examples such as one user stating that “everyone knows it was your app pinging” and another writing that the app “pings you because you walk past someone in the street.” These tweets suggested that the app was acting autonomously and had its own agency through personalization and, in the case of “someone,” an indeterminism.

Additional instances of the app “telling” (LogDice: 7.63) recounted personal experiences and fewer reported hypothetical scenarios. Examples included one user stating that the app “tells” them they “have to isolate” from 10 days after the initial encounter date. Another question asked why the app was “telling” them “to isolate for 14 days” when they believed it was 10 days instead. Overall, the app was actively presented in these scenarios. Therefore, should someone be affected by COVID-19, the app may be more likely to be presented actively.

Hypothetical instances questioned the legitimacy of the app, such as one user hypothesizing why other individuals were self-isolating when they had no symptoms because “an app told you to.” This presents the app as an implicated social actor. This could be seen to lessen the impact of the app, although presented actively, and may doubt the functionality of the system as a whole. Other active presentations that implied that the app had social agency removed the idea of instructing people to self-isolate. For instance, one author tweeted about the app “is creating a notification that has been stuck” on their screen for a long time. The idea that the app was “creating” a notification may further position it as a social actor. Instead of using the verb “notify,” the author word class converted this to the noun “notification,” using it in conjunction with a more personalized verb, “create.” Therefore, this clearly indicated agency and placed responsibility on the app to self-regulate through the agency metaphor.

The authors using “do” (LogDice: 5.16) discussed the app’s failed expectations. An example included one user writing that, despite their partner testing positive, “the so called world beating app didn’t alert [them].” This active construction indicated that the app was perceived to be responsible for their safety.

**Table 7:** Top 18 words ranked by collocational strength of “app” + one verb to the right (R1) in January and February 2021.

Rank	Collocate	Frequency	Collocate frequency	logDice
1	ping	7	455	7.98
2	tell	23	3357	7.63
3	cost	4	323	7.4
4	notify	4	385	7.29
5	say	18	4011	7.05
6	state	3	366	6.91
7	use	24	6766	6.77
8	work	22	7093	6.58
9	alert	7	2050	6.52
10	show	4	1119	6.38
11	be	189	80,974	6.25
12	keep	5	2083	6.01
13	seem	3	1221	5.88
14	have	43	30,674	5.5
15	need	7	4738	5.47
16	do	26	23,295	5.16
17	track	3	2653	4.98
18	store	3	2838	4.9

#### Passive Presentations in January to February 2021

When considering passive presentations, shown in Table 8, many tweets released in January and February 2020 were concerned with an individual being literally or hypothetically instructed by the app, for example, “ping” (LogDice: 8.90). Being “pinged by the app” was “as reliable as a handbrake on a canoe.” According to another user, they had to isolate for 10 days after they “got pinged by the app.” Other collocates such as “alert,” “told,” and “isolate” also followed similar patterns. This culminated in Twitter users potentially seeing the app as exemplifying unreliable government handling of the COVID-19 pandemic, shifting responsibility from the app to these organizations.

The app was also presented passively in conversations about its producers. For example, one tweeted that they resided and worked in an area where the infection rate was high, yet “the app has been triggered once in its 4/5 months existence.” Although this tweet presented the app passively, it placed the blame on the creators of the app, without even mentioning them, applying a reverse-exclusion strategy.

**Table 8:** Top 4 words ranked by collocational strength of “by the app” + one verb to the left (L1) in January and February 2021.

Rank	Collocate	Frequency	Collocate frequency	logDice
1	ping	7	455	8.9
2	alert	3	2050	5.57
3	isolate	3	3137	4.96
4	tell	3	3357	4.86

### Period 4: Later Months (March-June 2021)

#### Active Presentations in March to June 2021

Between March and June 2021, a total of 740 active presentations of “app” were found in the data set collected, as shown in Table 9, numerous presentations of which presented the app as a social actor.

One of the strongest collocates from these months, “provide” (LogDice: 7.22), described the app as helpful. An example of this was a tweet that stated that the app “provides anonymous information including risk alerts by postcode, a symptom checker, and test booking,” which came from a devolved local NHS Twitter account. The app was presented as a social actor, supporting the idea of system confidentiality and positively evaluated. Until now, when the app had been actively portrayed, it had usually been negatively connotated. However, this was not the case for all instances of “provide,” with other examples including one user that questioned why the app did not “provide update information” about local infection levels, whereas another user stated that “the app provides little to no information,” thus indicating dissatisfaction with the app’s performance.

Similar to most “provide” occurrences, “help” (LogDice: 5.22) was mainly seen in advertisements from devolved NHS Twitter accounts. In these cases, tweets contained constructions such as the app “helps stop the spread of the virus.” Therefore, this presented the app as having a positive impact on society.

Although not a significant enough collocate to meet the minimum threshold, authors used “tell” in conjunction with “be,” when discussing the app. Instances included that the app was “telling [them] 10 days from the 26th instead 20th,” and another wondering how long they needed to isolate for, particularly if it was “just the 2 [days] that the app is telling [them].” Both of these examples could be categorized as a query about the lack of clarity that the app reflected as the rules about self-isolation were changing. As both constructions showed the app to be active, this not only added to the evidence of the app being presented as a social actor but also contributed to the discourse surrounding questions over the functionality of the app itself.

Authors used “have” (LogDice: 4.09) to present the system in an active way, with examples of tweets including “not only has the app failed me [...] it has created a problem for me,” indicating that responsibility is attributed to the app. Another interesting presentation discussed the app as only guidance, as it “has no legal force.” Here, the app is presented actively, yet the content of the structure could be argued to mitigate or remove social agency. This suggested a decrease in the system’s responsibility and control.

**Table 9:** Top 6 words ranked by collocational strength of “app” + one verb to the right (R1) in March, April, May, and June 2021.

Rank	Collocate	Frequency	Collocate frequency	logDice
1	provide	3	525	7.22
2	help	5	4271	5.22
3	be	68	80,974	4.78
4	work	6	7093	4.77
5	have	16	30,674	4.09
6	do	8	23,295	3.48

#### Passive Presentations in March to June 2021

Owing to the small number of passive presentations (5/745, 0.7%) from March to June 2021, collocation analysis would not be meaningful. However, upon manual inspection, these constructions were concerned with scenarios that did not involve the tweeting authors. For instance, one discussed a friend who “has been told by the app to stay in for 3 days.” These tweets foregrounded the importance of the experience of the public by genericizing indetermining and backgrounding the app.

### Period 5: “Pingdemic” (July 2021)

#### Active Presentations in July 2021

There were 374 active occurrences of “app” in the “NHSCovid19App” data set and a further 5734 occurrences in the “pingdemic” data set (total 6108/6230, 98.04%). The collocations are listed in Table 10.

The strongest collocate was “disagree” (LogDice: 10.39). However, upon manual inspection, this was a headline that had been quote-tweeted multiple times. The variations in the headline read “U.K. Leaders Hail a Return to Normal; Their Phone App Disagrees” and “Britain’s contact-tracing phone app disagrees, telling huge numbers of people to self-isolate.” The idea that the app disagreed with powerful human entities exemplified personalization. Despite this coming from only 2 sources, the high number of shares indicates that others engaged with the idea.

Another strong collocate was “send” (LogDice: 9.38), in reference to the app sending a total of approximately 600,000 notifications to self-isolate. One tweet stated that the app “sending too many spurious notifications will reduce compliance.” In this instance, the author presented the app as an active social actor because of the cause-and-effect relationship between the app and the members of the public, further implicating the app as an agent of change, showcasing it as a perceived responsible actor by the users.

The recorded resurgence of “ping” (LogDice: 8.54) in July 2021 is likely due to a new blended term for the increase in exposure notifications. In these instances, the app was presented actively, as performing actions ranging from matter-of-fact reporting (“NHS Covid app pinged 600,000 more people”) to the nonsensical (“Every time a Covid app pings Boris Johnson loses one of his wingdings”). In each of these occurrences, the app was still presented as having agency and being a social actor through personalization, and hence depicted as causing frequent disruptions.

One occurrence where the app was presented as actively, “pinging” expressed disdain toward the members of the public who “self isolate when the app pings [them] even though [they] don’t have to,” hence suggesting that they “will blame the government for [their] own decisions.” Despite the active presentation of the app, its impact as a potential social actor was mitigated through the author’s sarcastic tone. Arguably, members of the public who use the app should be accountable for their own actions rather than blaming the app. Other tweets appeared to support this view, such as one stating that “it’s not a pingdemic” as the app was “pinging ppl correctly,” and another that detailed the app was “pinging” because “it is doing its job.”. All these instances illustrate different ways in which responsibility can be attributed to entities other than the app.

“Tell” (LogDice: 9.15) once again revolved around instruction to self-isolate. One user wrote about how the app “is telling people to self-isolate” because of higher infection rates. This contrasted with other experiences, such as another user asking whether the app can “tell” them when they are “supposed to have been near an infected person.” Another user wrote “the app told something like 700,000 to isolate,” which resulted in allegedly instructing supermarket staff to isolate as they had mobile phones at work. This presented the app as a social actor, and perhaps as if it had a humanlike agency through personalization. Active presentation of the app was clear in these cases, as it demonstrated the system’s capacity to instruct, thus having a social impact.

The app caused disruption through many other active presentations. “Wreak” (LogDice: 6.52) was used when users said the app “wreaks havoc.” Similarly, “cripple” (LogDice: 5.70) featured in constructions like the “app cripples Britain.” In addition, “threat” (LogDice: 5.50) featured in a tweet stating that the app “threatened to bring parts of the economy to a standstill.” All these constructions presented the app as destructive and capable of creating harm, thus being responsible for social disruptions.

**Table 10:** Top 20 words ranked by collocational strength of “app” + one verb to the right (R1) in July 2021.

Rank	Collocate	Frequency	Collocate frequency	logDice
1	disagree	76	96	10.39
2	send	45	451	9.38
3	tell	57	1535	9.15
4	be	845	47,226	9.14
5	have	165	12,256	8.59
6	ping	58	3351	8.54
7	do	93	6490	8.53
8	beg	19	53	8.43
9	work	36	1752	8.39
10	fail	13	478	7.57
11	delete	16	1057	7.54
12	install	10	64	7.49
13	start	13	917	7.32
14	cause	19	2259	7.28
15	alert	10	585	7.13
16	say	19	2778	7.1
17	use	12	1352	6.98
18	force	9	672	6.92
19	go	15	2327	6.91
20	design	7	223	6.86

#### Passive Presentations in July 2021

Passive constructions were more frequent in July (122) than previously (5). The collocational strength of these is shown in Table 11. However, due to the greater volume of tweets in this part of the discourse, this was proportionally lower than that in September 2020. “Ping” (LogDice: 8.30) showed users speaking hypothetically once again. One user questioned how society would cope “if everyone pinged by the app asked for a PCR test,” while another user stated that “if you get pinged by the app you shouldn’t need to self isolate.” This may suggest that the focus was on the humans affected by the app rather than the app itself. This seemed to limit the system’s social agency through the background. Similarly, collocates “alert,” “contact,” and “isolate” were found in tweets surrounding with the same idea.

Conversely, another strong collocate, “cause” (LogDice: 6.98), was used differently. Although the app was seen to “cause” damage and chaos during the “pingdemic,” in active constructions, the passive presentations removed agency from the app. Examples included one user writing that “staff shortages have NOT been caused by the App” and another stating that the United Kingdom government was to blame, hence “it’s not a ‘pingdemic’ caused by the app.” Finally, another tweet built on this and criticized the media outlet The Daily Star for “adopting the right-wing press’s line that the ‘pingdemic’ is caused by the app.” This not only removed grammatical agency from the app but also mitigated its social agency by making other agencies appear more responsible.

**Table 11:** Top 6 words ranked by collocational strength of “by the app” + one verb to the left (L1) in July 2021.

Rank	Collocate	Frequency	Collocate frequency	logDice
1	ping	33	3351	7.02
2	alert	5	585	6.82
3	contact	6	1084	6.19
4	cause	9	2259	5.72
5	isolate	4	2063	4.68

### Summary of Results

These results suggest that the app was presented in a predominantly active way (97.43% of occurrences—13,879/14,245 constructions), although some active presentations gave the app more social agency than others did. Approximately 100 participants carried less agency by mitigating activity in either verb constructions or other contextual information. This indicates that the app was presented as a social actor in approximately 96.73% (13,779/14,245) of the cases. This examination showed that the 13,779 active presentations, where the app constitutes a social actor, can be split into 5 broadly recurring themes: app informing (21.47%), app instructing (15.33%), app giving permission (9.1%), app disrupting (5.02%), and app functioning or not functioning (49.07%; Table 12). To answer our research question, the discussion will elaborate on the links between these constructs and the relationship between what is present in the discourse and what is present in the literature.

**Table 12:** Comparison of the percentage of each theme found when the app is presented as a social actor.

Theme	Values (%)
Informing	21.47
Instructing	15.33
Giving permission	9.1
Disrupting	5.02
Malfunctioning	49.07

## Discussion

### Overview

As mentioned earlier, our analysis showed that the app was presented actively as a social actor on approximately 96% of occurrences and unearthed 5 main categories for these active presentations: informing, instructing, providing permission, disrupting, and functioning. These categories revealed the personalized and independent decision-making role of the app. As for passive presentations, instances of backgrounding, where developers and the public were foregrounded, obscured the app’s agency, potentially reflecting a small number of Twitter users’ beliefs about the app’s role and responsibility. This section explores the implications of these results.

### Principal Findings: Trends of Active Agency

#### Overview

Through the analysis of transitivity in the 14,425 concordance lines considered, the collocations of “app” and “by the app” and CDA-informed analysis of agency and responsibility, underpinned by SAR, we identified 5 main categories that the active presentations of the app fall into: informing (21.47%), instructing (15.33%), providing permission (9.1%), disrupting (5.02%), and functioning (49.07%). The first 4 categories show the app to be personalized [27] and to make decisions independently [24]. Meanwhile, functioning included instances of the app acting autonomously but also simply functioning as intended or designed to do. In addition, this category included tweets where the app was functioning appropriately, but also contained tweets where it was presented as not functioning as desired. This may explain the large percentage of tweets in this category.

#### Informing

The app was presented actively (21.47% of occurrences) when providing information to its users through “saying” and “pinging.” This happened especially at the start of the discourse, as the app was “saying” information that was difficult to understand (LogDice: 8.07). Similarly, users complained about the app informing them (41/64, 64% occurrences), determining a trend that followed other areas of discourse. An example of this was in the “early months” part of the timeline, where the app communicated about the status of their self-isolation period (LogDice: 8.58). In this sense, the idea of presenting information linked to the findings of the study by Williams et al [44], while also pointing out that the app proving information to users was deemed a core responsibility of the app, hence the questions and negative reactions when the app that “failed.”

The app was presented actively informing also through the surge in “ping,” a frequent collocate of “app” between January and February 2021 (LogDice: 7.98). The tweets containing these collocates depicted the app as acting autonomously, once again leaning toward personalization. Although some instances later in the discourse presented the app as providing useful information, most presentations still remained negative when it came to the information given—or not—to users (LogDice of “provide” in March-June 2021 being 7.22). “Ping” clearly continued being a verbal trend into July 2021, with many tweet authors discussing the impact that the app informing them of a COVID-19 positive or isolation status (LogDice: 8.54). This is complemented by strong collocations of “tell” (LogDice: 9.15) and “say” (LogDice: 7.10). Therefore, it could be inferred that the app’s active presentation, using “ping,” and the perception that it might have provided incorrect information contrasted with the rationale for having a decision-making algorithm in the first place [4].

#### Instructing

The app was also seen as actively providing instructions to users and the wider public (15.33% of occurrences). “Tell” was a frequent collocate of “app” throughout the discourse (LogDice scores of 7.90, 8.55, 7.63, and 9.15), and users presented this as the app instructing them to take action, most notably, to self-isolate (232/270, 85.9% occurrences). At the beginning of the discourse, 30 of 52 occurrences were hypothetical, likely due to the app being newly launched. Twitter users also questioned the instructions provided by the app (12/52, 23% occurrences). This imperative tone continued in the final months of 2020, with users stating that the app instructed them to self-isolate (44/61, 72% occurrences). However, what became more apparent in this section of the discourse was that, although the app was presented as a social actor through personalization, the impact of this system was ridiculed through humorous additions to tweets or sarcastic tone (9/61, 15% occurrences). This likely softened the instructional impact that the app had, while still presenting it as a social actor.

The app continued to be presented as actively instructing in later parts of the discourse too, with direct first person accounts of experiences when the app “is telling” users (LogDice: 9.15), as well as reports of hypothetical scenarios (14/23, 61% occurrences). These constructions exhibited human-level agency through personalization. The other constructions seen in July 2021, such as those that contain “wreak,” “cripple,” and “threat,” implied equally a significant level of agency as if the app’s instructions could only result in negative consequences, although this will be explored in more detail in the “disrupting” section. The presentation of the app in this way intersected with concerns about the merging of algorithmic and human agency [8,9,39,88] because the app is presented as performing the job of a human. In particular, the app was featured in constructions where users were frustrated with its instructions and users or their lack, ultimately providing insights into the agency of the algorithm’s perceived role and responsibility.

#### Giving Permission

Although less common than the previous 2 categories, the app was also presented actively when providing users or general public permission (9.1% of occurrences). This is most prominently seen in users stating that the app is “allowing.” More present at the start of the discourse (LogDice: 7.35 for September 2020 and 6.73 for October-December 2020), due to the questions being asked about the app, this was less frequently discussed as time passed. Sets of tweets in the discourse pointed to the app providing permission. For example, the app “gives” notification of self-isolation periods at the end of 2020. This recalled some permission concerns found by Dowthwaite et al [16].

It could also be argued that “need,” a strong collocate at the beginning of the discourse (LogDice: 7.50), intersected this theme and “functioning.” The idea that the app needed to provide permission to humans was an occurrence of personalization, providing further insight into the idea that the app was not only given agency but also showcasing its necessity to process information systematically.

#### Disrupting

The app was presented as disrupting users’ lives through active constructions. This is seen early in the discourse, when users commented on the app making disruptive decisions autonomously, such as turning off contact-tracing functionalities (88/495, 17.8% occurrences). This continued throughout the discourse, with users describing the problems the app has caused them. However, most tweets that suggested the app was actively disrupting the lives of the public appeared more toward the end of the sampled period, when the “pingdemic” occurred (456/845, 54% occurrences). Examples of these included instances when the app was defined as “wreak havoc,” “cripple Britain,” and “threaten the economy.” This relates to the idea that the system failed to meet the needs and expectations of users [15,17]. It also supported the findings of Lamanna and Byrne [37] and Riegler [38], according to whom humans could be perceived as “at odds” with the decisions made by this system.

#### Functioning

One final category (49.07% of occurrences) was the app being presented as independently undertaking (or attempting to undertake) functional activities that were integral to its running. Tweets in the later months of 2020 stated that the app had a job, a clear personalization (LogDice: 7.02). Tweets in this discourse indicated that one of the intended primary functions of the app was to help or assist users. When this was perceived as not happening, the app was not fulfilling its (supposed) rationale for existing. This is particularly prevalent when the app was said to not be “helping,” at the start of 2021 (LogDice: 5.22), and perceived as failing to keep users safe. The fact that Twitter authors saw the app as being responsible for their safety and welfare showed its prominence and influence as a social actor, similar to the findings of Kent [15] and Mbwogge [17]. In the later parts of the discourse, the app was occasionally presented as having limited legal power or obligation over users (48/283, 17% occurrences), providing insight into how the app was perceived as responsible for its users.

During the “pingdemic” part of the discourse, the app was said to “send” (LogDice: 9.38) many notifications, suggesting that the app was designed for this. Many of these tweets indicated that, although the app was not necessarily instructing users, it encouraged noncompliance with too many notifications. This recalled the findings from the follow-up study by Pepper et al [2]. In addition, it may also indicate that the app “pinging” was perceived as more invasive than simply “saying.” Despite users wanting the app to function properly, they appeared to find “pinging” overbearing.

In addition, personalization was observed when looking at the app’s perceived functionality. For example, the collocate “think” (LogDice: 7.24), seen throughout the discourse, regularly presented the app as stating something that was incorrect (30/39, 77% occurrences). This was related to the app not working as perceived by the Twitter user. This indicated that the app was perceived as having the capacity to think or act autonomously, leading to the opposition of system use [40,41,88].

### Principal Findings: Trends of Passivization

Instances of backgrounding were found throughout the discourse. Examples of this included the way in which the developers of the app (Serco, the NHS, and the UK government) were foregrounded, especially at the start of the discourse, and how the public, affected by COVID-19 and isolation requirements, became a focus over time. This meant that the app was backgrounded according to the principles of SAR [27], with its agency obscured [26]. The app was still discussed negatively in these constructions, despite not being an overt social actor, due to its reduced agency. This presentation intersected part of the work outlined by Feier et al [11], who suggested that decision-making algorithms may deflect blame from more responsible players. In addition, this portrayal may be a reflection of the perceived attitudes of Twitter users; some (14/42, 33% occurrences) believed that, while the app played an important role, the responsibility remained with the developers of the app or with the humans that used the app at their own discretion. The removal of the agency diluted the impact [31]. That said, the proportion of passive presentations of the app was very small (approximately 4% of all constructions) in comparison with active presentations.

However, considering verb choices, such as “tell” and “cause,” passive constructions were still present. Thus, a small portion of tweets (32/366, 8.7% occurrences) using passive constructions appeared to imply that the app still has some agential power, which may be labeled as agency metaphors [32]. Such agential power could be considered as impacting the app; hence, the app was still deemed to have some responsibility for processing information.

### Limitations

With a corpus of 118,316 tweets, it would have been practically impossible to manually examine each [89]. Hence, CL was used to filter the data set collected and identify relevant potential social actors through the analysis of the keyword “app” and its 14,245 collocates in the corpus, which were examined through concordance grids and LogDice. This methodological approach was intended to mitigate this issue of infeasibility.

Another limitation posed by CDA was subjective biases, impacting the interpretation of instances of sarcasm and humor, especially those that were less explicit. In this sense, this challenge is not new to researchers [90,91]. Nonetheless, the combination of CDA with computationally aided techniques was intended to reduce the impact of this difficulty and may benefit future research.

As “app” was the key-term searched, this work disregarded most instances where exclusion masked the app in constructions and the actual word did not feature. Nonetheless, this could constitute an interesting future research focus that encompassed explicitly excluded constructions. In addition, the system may have been discussed in tweets without specific reference to “app.” Although other social actors replacing “app” would be hard to find a large corpus, a good starting point may be synonyms of “app” in this specific context, such as “(information) system,” “application,” “tool,” “program” or “software.” Similarly, related field-specific words may also offer relevant research insights, such as “functionality,” “function(s),” “operation(s),” “spread(ing),” “track(ing),” or “trace/tracing.”

Due to the brevity of Twitter discourse, which is limited by a 280 character limit, the app may have been presented actively to facilitate conciseness. For example, “the app told me to isolate” (26 characters) contained 6 fewer characters than “i was told to isolate by the app” (32 characters), which could have been an equally valuable semantic alternative. Such a possibly increased number of active presentations is likely to have affected the number of times the app was presented clearly as a social actor. Consequently, future work may involve examining other social media or text-sharing platforms that do not limit characters in posts or content to see if the most active agential presentations are comparable.

### Conclusions

According to our CL-, CDA-, and SAR-based examination of agency and transitivity in tweets containing the word “app,” published between September 2020 and July 2021, Twitter users presented the NHS COVID-19 App as a social actor and with a clear sense of social agency, addressing our first research question concerning how society had been impacted by the deployment of this system (ie, the NHS COVID-19 App).

Specifically, the app was predominantly presented actively by Twitter users in 96% of the cases, using various techniques, most notably personalization, but also including determination, agency metaphor, and genericism. Indeed, we found that these active presentations, which implied social agency, primarily conveyed the idea of app informing (21.47%), instructing (15.33%), providing permission (9.1%), disrupting (5.02%), and functioning or failing to (49.07%).

The app was also presented passively on occasions (approximately 3%), although this decreased as the discourse continued, reaching a maximum impact of 4% in September 2020 and decreasing to a minimum of 2% in July 2021. In such instances, the app was often backgrounded to make the developers or operators of the app more apparent or responsible. On occasion, the focus was on the members of the public affected by the app malfunctioning rather than the app itself. Comparable instances, when the impact of the app as a social actor was limited, the app was presented actively but simultaneously ridiculed.

The implications for this study, with regard to our second research question concerning the app’s perceived responsibility to process information, are that Twitter users presented the app as responsible for their own welfare through various active presentations, especially when the app instructed them or provided permission. According to the tweets examined, the perceived responsibility to process information remained in the app throughout the discourse. Such a perception was especially pronounced when significant events prompted further questioning of the app’s capabilities (ie, during the app’s launch in September 2020, the second lockdown in January 2021 and the “pingdemic” phase in July 2021).

In addition to offering insights into web-based responses to this specific event, this contribution holds the potential for broader implications in the context of decision-making algorithms. Although the disruption caused by the pandemic has waned in the United Kingdom, the findings of this study shed light on how the public might respond to forthcoming decision-making algorithm interventions. This insight is particularly valuable in the context of health care or digital contact-tracing initiatives, shining light on barriers to adoption. Therefore, even in a postpandemic world, the findings of this study remain important.

Overall, this study has provided insights into how social agency communicated via social media public discourse dealing with algorithmic-operated decisions when the AI agency behind those information systems is not openly disclosed. Such a relationship was exemplified by that between the NHS app, grammatical agency, and social agency, building on existing work on the social agency of decision-making algorithms [33,36,37,40]. Therefore, our study contributes to the investigation of the social impact of the NHS COVID-19 App, in particular, showcased through the combination of CL and CDA underpinned by SAR. Briefly, our research argues that the views expressed on social media indicate that the app was presented as having a perceived high level of responsibility for the welfare and safety of its users according to tweets that explicitly referred to the app.

## Abbreviations

AI: artificial intelligence
CDA: critical discourse analysis
CL: corpus linguistics
NHS: National Health Service
SAR: social actor representation

## Data Availability

All data generated or analyzed during this study are included in this paper and its supplementary information files.
